# Ultrapotent neutralization of *Bacillus anthracis* toxin by a human antibody via blockade of PA oligomerization

**DOI:** 10.1371/journal.ppat.1014523

**Published:** 2026-08-14

**Authors:** Qi Wang, Ting Fang, Zhenwei Song, Xiaoyan Huang, Sijun He, Tianfu Li, Jianmin Li, Xiangyang Chi, Pengfei Fan, Changming Yu

**Affiliations:** 1 School of Pharmacy, Nanjing University of Chinese Medicine, Nanjing, Jiangsu, China; 2 National Key Laboratory of Advanced Biotechnology, Academy of Military Medical Sciences, Beijing, China; Children’s Hospital Boston and Harvard Medical School, UNITED STATES OF AMERICA

## Abstract

Anthrax lethal toxin is a key virulence factor of *Bacillus anthracis*. However, non-canonical epitopes of protective antigen (PA) and alternative neutralization mechanisms beyond receptor blockade or prevention of proteolytic cleavage remain underexplored. Here, we characterize a fully human monoclonal antibody, 22F1, with ultrapotent toxin neutralization in vitro (IC_50_ = 0.027 nM) and in vivo (molar ratio 1:7.5–15). Unlike known PA antibodies, 22F1 selectively binds the post-cleavage form PA63, not full-length PA83, due to shielding of the epitope by PA20. Cryo-EM reveals a novel conformational epitope spanning domains D1′, D2, and D3. 22F1 does not block receptor engagement, PA proteolytic activation, or lethal factor binding. Instead, its epitope lies near the inter-protomer interface; binding of a single 22F1 Fab to any PA63 protomer exerts a dual steric hindrance effect, simultaneously preventing additional Fab binding to adjacent protomers and blocking PA63 oligomerization. This “single-hit” mechanism enables one antibody molecule to efficiently block prepore formation, achieving high potency. Our findings advance anthrax antitoxin research and provide a paradigm for targeting other proteins with similar activation–oligomerization–pore formation mechanisms.

## Author summary

Anthrax remains a major threat, yet non-canonical PA epitopes and alternative neutralization mechanisms are underexplored. Here we introduce 22F1, a fully human antibody that potently neutralizes anthrax toxin. Unlike conventional antibodies, 22F1 binds selectively to the cleaved, activated form of PA—not full-length PA83—due to epitope masking by PA20. Cryo-EM reveals a novel epitope spanning PA63 domains D1′, D2, and D3. 22F1 does not block receptor binding, LF attachment, or PA cleavage. Instead, a single 22F1 Fab docking onto any PA63 protomer generates dual steric hindrance: it prevents additional Fab binding to adjacent protomers and blocks prepore formation. This “single-hit” mechanism explains its exceptional efficacy. Our work provides a potent anthrax antitoxin candidate and establishes a new paradigm for targeting proteins that undergo activation–oligomerization–pore formation.

## Introduction

*Bacillus anthracis*, a gram-positive, spore-forming bacterium and the causative agent of anthrax, remains a significant threat to public health and biosecurity because of its long-lived spores, potential for aerosol dissemination, and high mortality in systemic disease [1,2]. Its virulence is governed by two plasmids: pXO1, which encodes the tripartite exotoxin components protective antigen (PA), lethal factor (LF), and edema factor (EF), and pXO2, which directs synthesis of the anti-phagocytic poly-γ-D-glutamic acid capsule [3]. PA is the central binding and translocation component of anthrax toxin, mediating the delivery of LF and EF into the host-cell cytosol. There, LF—a zinc-dependent metalloprotease—cleaves mitogen-activated protein kinase kinases, while EF—a calmodulin-dependent adenylate cyclase—elevates intracellular cAMP. Collectively, these activities disrupt immune signaling, impair barrier integrity, and drive vascular dysfunction and rapid disease progression [4].

The pivotal role of toxin in anthrax pathogenesis has strongly shaped therapeutic development. Antibiotics effectively eliminate replicating bacilli but do not neutralize pre-existing toxins, which may continue to drive injury even after bacterial clearance—a limitation that has spurred interest in passive immunotherapy [5]. Over the past two decades, antibody-based approaches have advanced from preclinical concepts to licensed countermeasures. Most antibodies developed to date target PA, owing to its essential and multifunctional role in toxin entry. Full-length PA (PA83) comprises four domains: domain 1 (D1, 1–258 aa) contains the furin cleavage site; domain 2 (D2, 259–487 aa) forms the transmembrane pore; domain 3 (D3, 488–595 aa) mediates oligomerization of PA63; and domain 4 (D4, 596–735 aa) is primarily responsible for binding to cell surface receptors TEM-8 (ANTXR1) and CMG-2 (ANTXR2) [6,7]. This modular architecture enables anti-PA antibodies to act through multiple, non-mutually exclusive mechanisms, including blockade of receptor binding, interference with proteolytic activation, disruption of oligomerization, prevention of LF/EF docking, and impairment of pore formation or translocation [8].

Early animal studies in the 2000s established that anti-PA monoclonal antibodies could confer robust protection when administered either prophylactically or therapeutically in spore-challenge models [9–17]. The FDA approvals of raxibacumab (2012) and obiltoxaximab (2016) marked the clinical translation of this concept. Mechanistically, even with comparable affinities, antibodies can exhibit markedly different neutralizing activities owing to the specific epitopes they recognize or the particular intoxication steps they block [18,19]. Building on this understanding, research in recent years has shifted focus from antibody discovery to epitope definition and mechanism‑driven optimization, further expanding the diversity of candidate antibody repertoires and neutralizing epitope maps, and reaffirming PA as a viable therapeutic target [18–21]. Moreover, beyond monoclonal antibodies, recent studies have increasingly emphasized the translational value of antibody engineering and combination strategies, driving the development of antibody cocktails and bispecific formats that target non‑overlapping PA epitopes to simultaneously block multiple steps of toxin entry [22–25].

Despite these advances, important gaps remain. Much of the field has focused on canonical neutralizing epitopes of the secreted PA precursor, whereas non-canonical PA epitopes remain comparatively underexplored. Direct side-by-side comparisons under standardized conditions are limited, and for several proposed modes of neutralization—particularly those involving oligomerization or prepore formation—experimental support remains incomplete. In this context, antibodies with atypical binding and neutralization profiles are of special interest, as they may reveal previously unrecognized vulnerable surfaces on PA and expand the mechanistic toolkit for therapeutic design.

Here, we revisit a previously described human anti-PA antibody, 22F1 [18], which displays an unusual binding and neutralization profile not fully explained by established mechanisms. We evaluated its neutralizing activity in vitro and in vivo, mapped its epitope on PA, determined the structural basis of antigen recognition, and investigated how antibody binding interferes with toxin function. Our results reveal a cleavage-dependent epitope on PA and an efficient neutralization mechanism, providing new insight into underappreciated therapeutic vulnerabilities of anthrax toxin and offering a framework for designing improved antibody‑based countermeasures.

## Results

### 22F1 binds post-cleavage forms of PA with a pattern distinct from that of known mAbs

**Fig 1:**
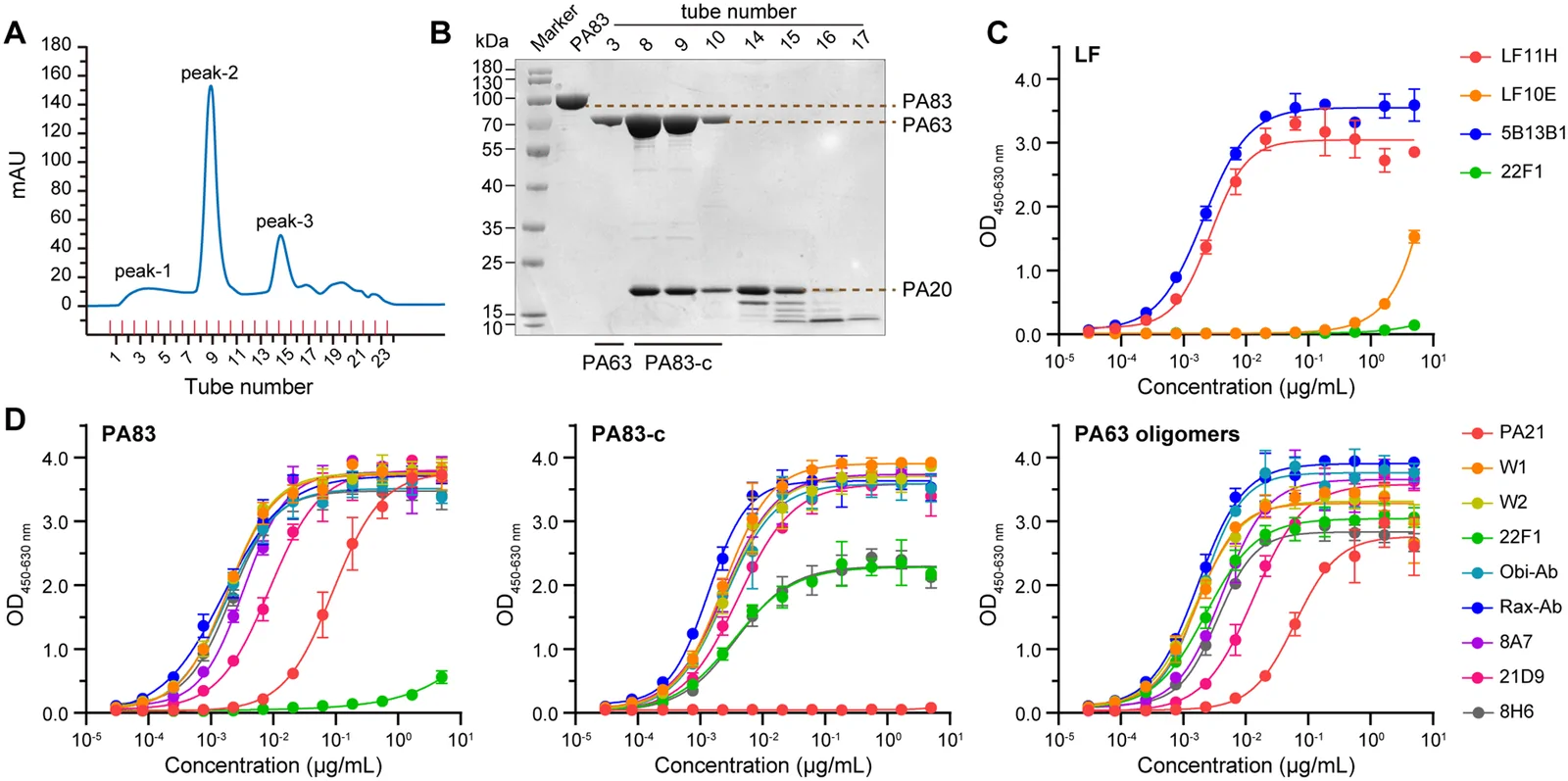
Binding profiles of antibodies against PA or LF. **(A)** Size-exclusion chromatography profile of trypsin-digested PA83 products on a Superdex 200 10/300 GL column, showing three major peaks. **(B)** Reducing SDS-PAGE analysis of the chromatographic peak fractions. **(C)** Binding curves of anti-LF antibodies to recombinant LF protein determined by ELISA. Data are presented as mean ± SD (*n* = 3). **(D)** Binding curves of anti-PA antibodies to PA83, PA83-c, and PA63 oligomers determined by ELISA. Data are presented as mean ± SD (*n* = 3).

To characterize the binding properties of 22F1, PA83 was subjected to in vitro trypsin digestion, and the resulting products were separated by size-exclusion chromatography, which yielded three main peaks (Fig 1A). Reducing SDS-PAGE analysis revealed that, in order of elution, these peaks corresponded to heptameric or octameric PA63 oligomers [26,27], a presumed PA63‑PA20 post‑cleavage complex (designated PA83-c), and PA20 (Fig 1B). Given the rapid dissociation of PA20 and subsequent oligomerization of PA63 following PA cleavage [28], and the observed coexistence of PA63 and PA20, the material eventually obtained from the PA83‑c peak is likely a mixture composed of PA63-PA20 complexes, PA63 monomers, PA20, and PA63 oligomers. Subsequently, 22F1 was compared with previously reported PA antibodies (PA21 [17], W1 [13], W2 [13], 21D9 [10,11], obiltoxaximab [29], raxibacumab [30], 8A7 [18], and 8H6 [18]) and LF antibodies (LF11H [31], LF10E [31], 5B13B1 [32]) by ELISA to assess the binding activity to various antigens. 5B13B1 and LF11H effectively bound LF, with EC_50_ values of 2.55 ng/mL and 4.54 ng/mL, respectively, whereas LF10E showed almost no binding to LF (Fig 1C and Table 1). All PA antibodies recognized PA63, albeit with a wide range of EC_50_ values (2.07–116.79 ng/mL) (Fig 1D and Table 1). Notably, two antibodies exhibited distinct recognition patterns: PA21 failed to bind PA83-c, whereas 22F1 displayed negligible binding to PA83. Although 8H6 and 22F1 did bind PA83-c, their binding activity was relatively weaker, as reflected by higher EC_50_ values and lower maximal optical density signals compared to the strong binders.

**Table 1:** Summary of binding and neutralizing activity of antibodies.

mAb	ELISA binding EC_50_^a^ values (ng/mL)				Toxin neutralization IC_50_^b^ values (μg/mL)
	PA83	PA83-c	PA63	LF	LT
PA21	106.29	NM	116.79	ND	NM
W1	1.97	2.42	2.18	ND	0.07
W2	2.08	2.35	2.21	ND	0.10
22F1	NM^c^	4.75	2.43	ND	0.004
obiltoxaximab	2.51	2.95	3.05	ND	0.17
raxibacumab	2.59	1.46	2.07	ND	>1.00
8A7	3.10	2.79	3.74	ND	NM
21D9	7.93	3.79	12.85	ND	0.01
8H6	2.62	6.75	2.75	ND	ND
5B13B1	NM	NM	NM	2.55	NM
LF11H	ND^d^	ND	ND	4.54	NM
LF10E	ND	ND	ND	NM	NM

^a)^EC_50_, the half-maximal effective concentration determined by ELISA; ^b)^IC_50_, the half-maximal inhibitory concentration measured by LT neutralization tests; ^c)^NM, not measurable (non‑binding or non‑neutralizing); ^d)^ND, not determined.

**Fig 2:**
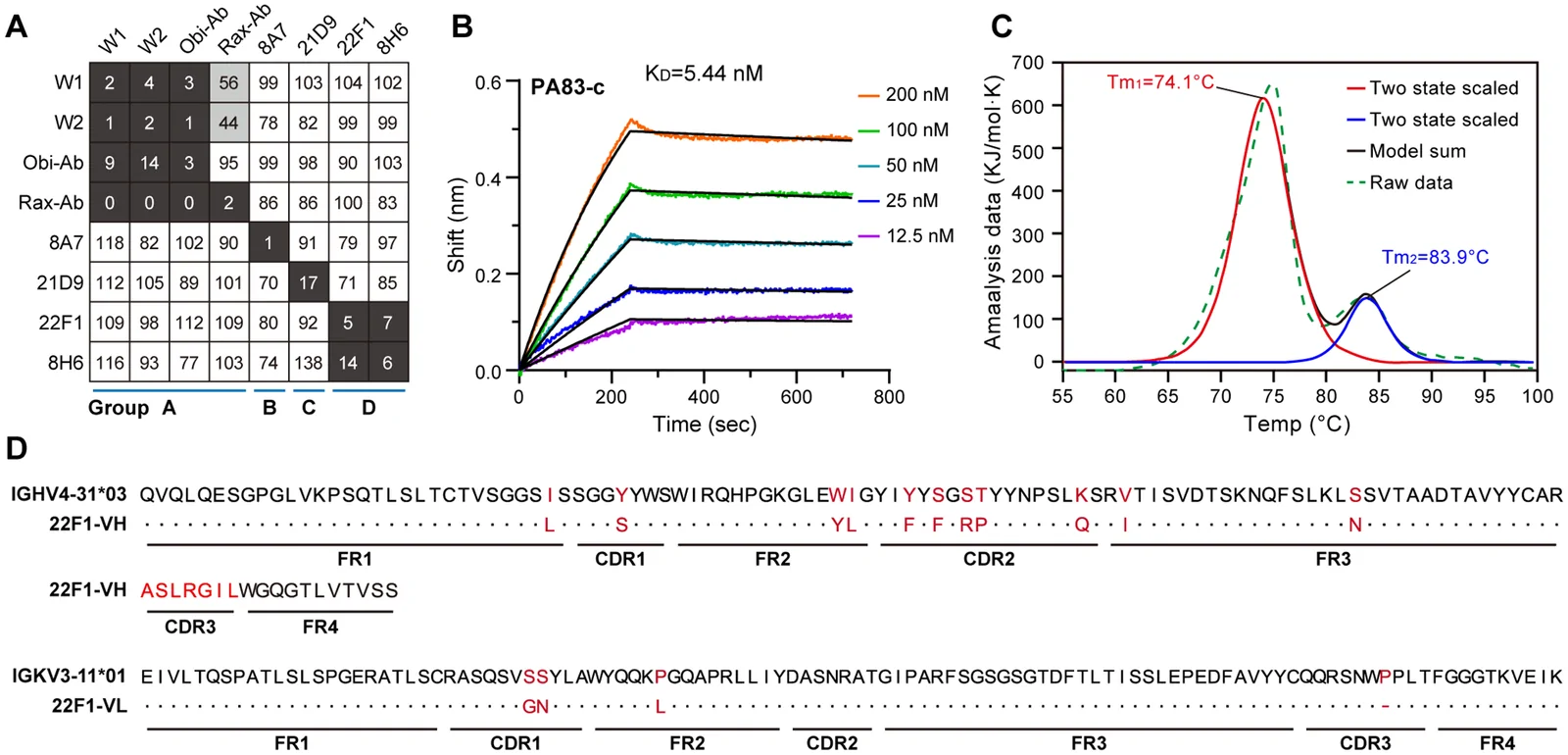
Epitope competition, thermal stability, and sequence analysis of 22F1. **(A)** Competition binding assay performed by ELISA. The level of competition is determined based on the relative binding percentage of biotinylated mAb in the presence or absence of the competing mAb: < 33.3%, strong competition (black background with white text); 33.3–66.7%, intermediate competition (gray background with black text); > 66.7%, no competition (white background with black text). **(B)** Binding kinetics of 22F1 Fab to PA83-c measured by biolayer interferometry (BLI). Five representative binding curves for PA83-c at concentrations ranging from 200 to 6.25 nM (2-fold serial dilution) are shown, and kinetic constants were calculated by curve fitting to a 1:1 Langmuir binding model. **(C)** Thermal stability of 22F1 measured by differential scanning calorimetry. Raw data were fitted using the two-state scaled model. **(D)** Amino acid sequence alignment of the 22F1 variable regions with the inferred germline genes. Residue differences are highlighted in red. Framework regions (FRs) and complementarity-determining regions (CDRs) are annotated using abYsis.

Next, competitive binding assays were performed with eight of the PA antibodies (excluding PA21, which exhibits a low binding ability to PA63) using PA63 as the antigen. Based on the competition patterns, the eight antibodies were classified into four groups: A (W1, W2, obiltoxaximab, raxibacumab), B (8A7), C (21D9), and D (22F1 and 8H6) (Fig 2A). Bio-layer interferometry was used to determine the binding kinetics of the 22F1 Fab to PA83-c. The results showed a low association rate but an extremely low dissociation rate, with an equilibrium dissociation constant (*K*_D_) of 5.44 nM (Fig 2B). Thermal stability was assessed by differential scanning calorimetry (DSC). The DSC profile of 22F1 exhibited two endothermic peaks with Tm1 = 74.1°C, Tm2 = 83.9°C, and Tonset > 60°C (Fig 2C), indicating good thermal stability. Comparison of the 22F1 variable region sequences to germline genes revealed a higher number of somatic mutations in the variable region of the heavy chain (VH; 11/106 amino acids) than in that of the light chain (VL; 3/111 amino acids) (Fig 2D).

### 22F1 exhibits ultrapotent LT neutralization activity in vitro and in vivo

**Fig 3:**
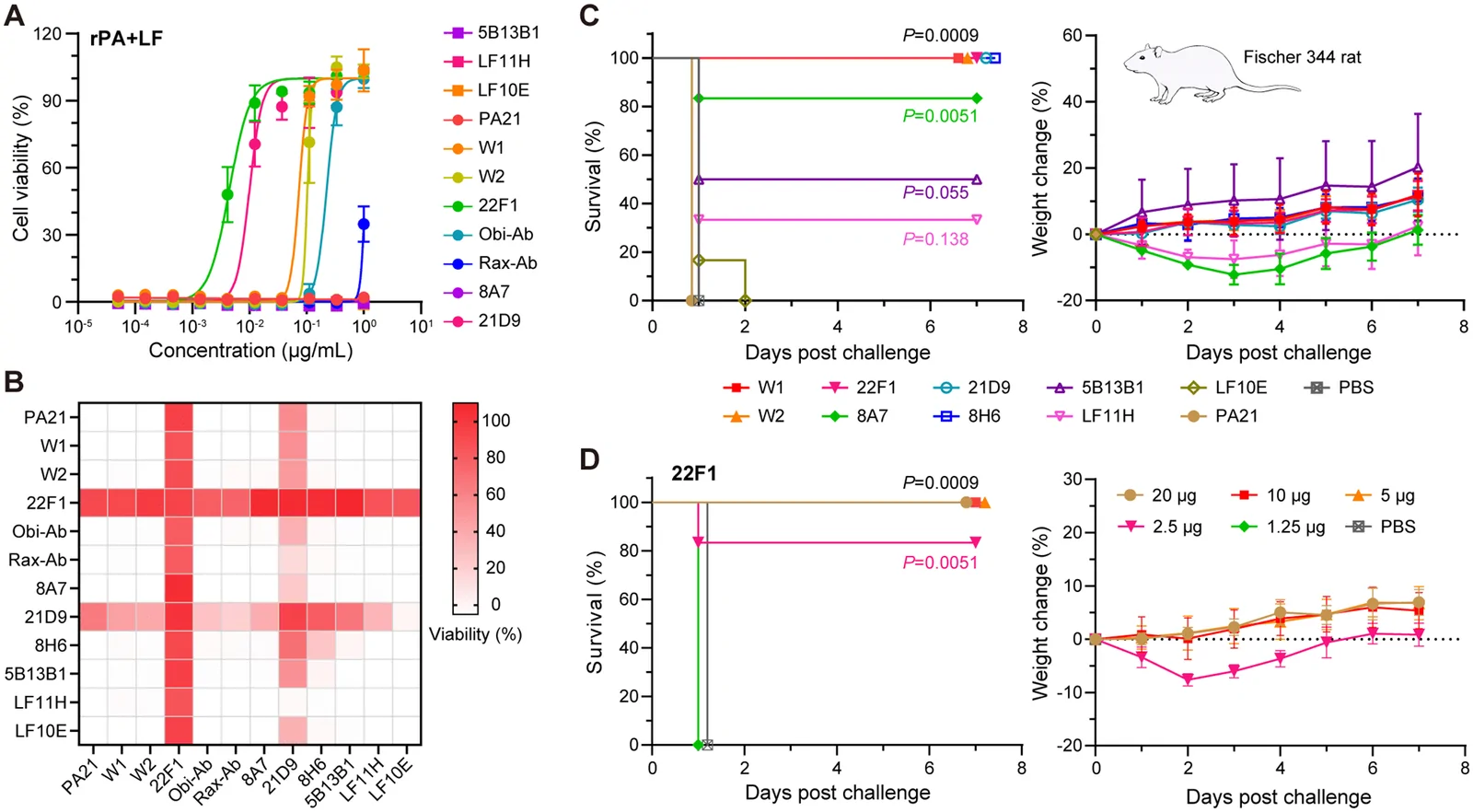
In vitro and in vivo neutralization of anthrax lethal toxin by antibody 22F1. **(A)** Neutralization activity of the indicated antibodies against lethal toxin (LT) in J774A.1 cells. Data are presented as mean ± SD. **(B)** Synergistic neutralization assay. Antibody pairs were each used at a final concentration of 10 ng/mL. **(C)** Survival and body weight changes of Fischer 344 rats (*n* = 6) after intraperitoneal injection of antibody (20 μg) mixed with toxins (PA 20 μg + LF 10 μg). Survival was monitored for 7 days. *P* values were determined by the Mantel–Cox log-rank test. *P < 0.05, **P < 0.01, ***P < 0.001. **(D)** Dose-dependent protection of 22F1. Rats received fixed toxin doses (PA 20 μg + LF 10 μg per rat) combined with 22F1 at 20, 10, 5, 2.5, 1.25, or 0 μg per rat (*n* = 6 per group). *P* values are the same as in (C).

To evaluate the neutralizing activity of the antibodies, their efficacy against anthrax LT was compared using the J774A.1 cell model. None of the three LF antibodies showed neutralizing activity (Fig 3A). Among the PA antibodies, 8A7 and PA21 lacked neutralizing activity; raxibacumab showed only weak activity; and the remaining five antibodies fully inhibited LT at high concentrations. Remarkably, 22F1 exhibited ultrapotent neutralizing activity with an IC_50_ of 0.004 μg/mL (≈ 0.027 nM), which was 2.5-fold more potent than that of 21D9 and 17.5- to 42.5-fold more potent than those of the other neutralizing antibodies tested (Table 1). Further assessment of synergistic effects among antibodies showed that none of the antibody combinations outperformed 22F1 alone in toxin neutralization (Fig 3B).

The in vivo protective efficacy of the antibodies was evaluated in the Fischer 344 rat model. All rats in the PBS control group succumbed within one day post-challenge. At a 1:1 mass ratio of antibody to PA, the five neutralizing antibodies provided complete protection, and rats in these groups showed no significant body weight loss (Fig 3C). In contrast, the non‑neutralizing PA antibody PA21 and the non‑neutralizing LF antibody LF10E failed to protect. Notably, despite lacking in vitro toxin‑neutralizing activity, antibody 8A7 conferred 83% survival (*P* = 0.005). The other two non‑neutralizing antibodies, 5B13B1 and LF11H, provided partial protection (50% and 33% survival, respectively), but these differences compared to the control group were not statistically significant (*P* = 0.055 and *P* = 0.138, respectively). Dose‑response protection experiments demonstrated that as little as 5 μg of 22F1 (corresponding to a molar ratio of 22F1:PA of approximately 1:7.5, given a PA challenge dose of 20 μg) provided 100% protection (*P* = 0.0009), with no significant body weight loss in rats; even at 2.5 μg, 83% protection was observed (*P* = 0.005) (Fig 3D).

### 22F1 has a unique neutralization mechanism

**Fig 4:**
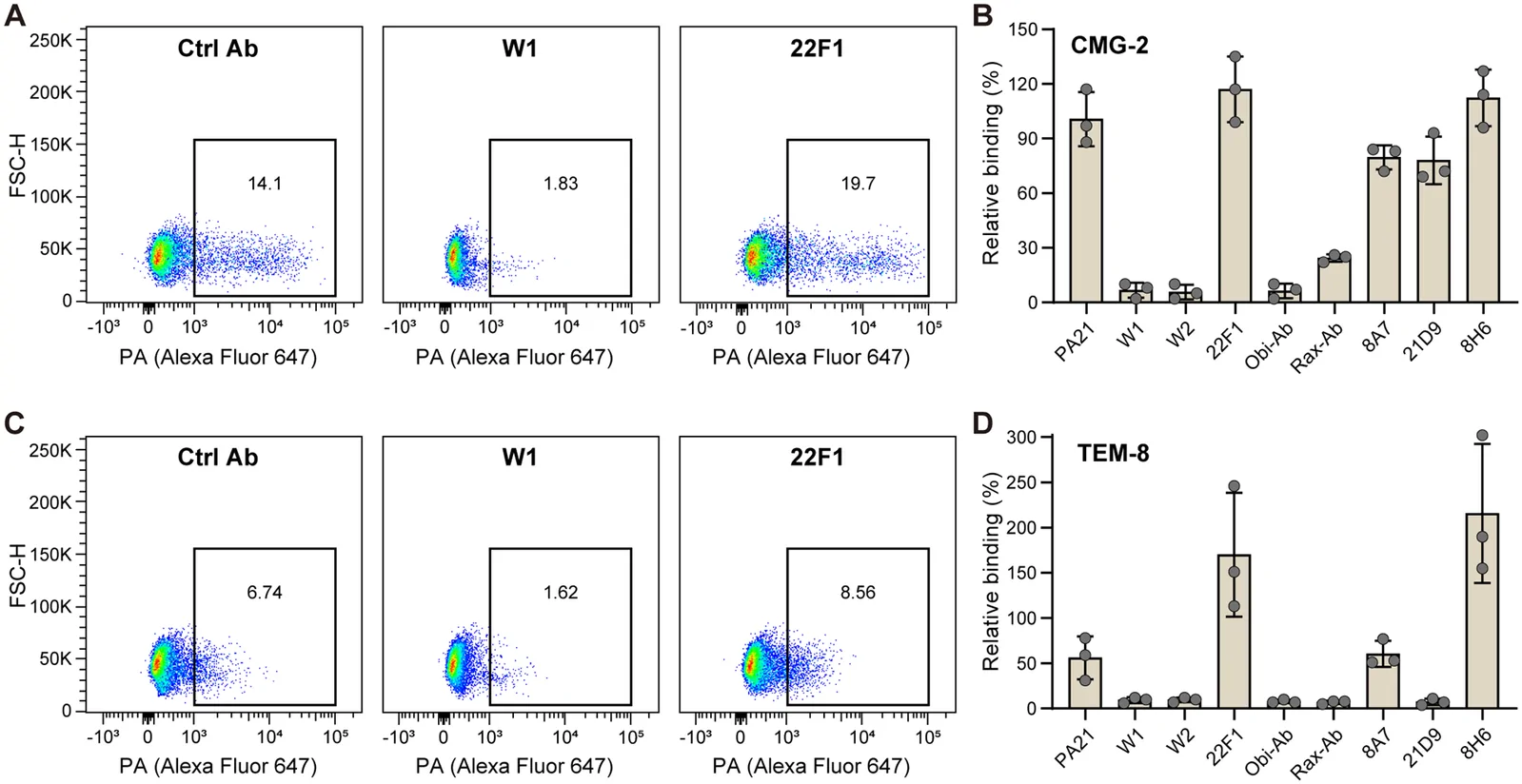
Antibody blockade of AF647‑labeled PA binding to CMG‑2 and TEM‑8. **(A, C)** Representative flow cytometry scatter plots of AF647‑conjugated PA83 (1 μg/mL) binding to HEK293T cells transiently expressing CMG‑2 (A) or TEM‑8 (C), in the presence of the indicated anti‑PA antibodies (4 μg/mL). 2G1, an anti‑Ebola virus glycoprotein human monoclonal antibody, served as an isotype control. **(B, D)** Quantification of relative binding (%) to CMG‑2 (B) and TEM‑8 (D), calculated as the percentage of AF647‑positive cells normalized to the isotype control. Data are shown as mean ± SD (*n* = 3).

To elucidate the potent neutralization mechanism of 22F1, systematic analyses were performed. First, flow cytometry was used to assess the ability of antibodies to block PA binding to cell surface receptors (CMG-2 and TEM-8). All four antibodies in competition group A effectively blocked PA binding to both CMG-2 and TEM-8 (Fig 4). 21D9, PA21, and 8A7 did not block PA binding to CMG-2 but completely or partially blocked binding to TEM-8. For 22F1, no blocking of binding to either receptor was observed.

**Fig 5:**
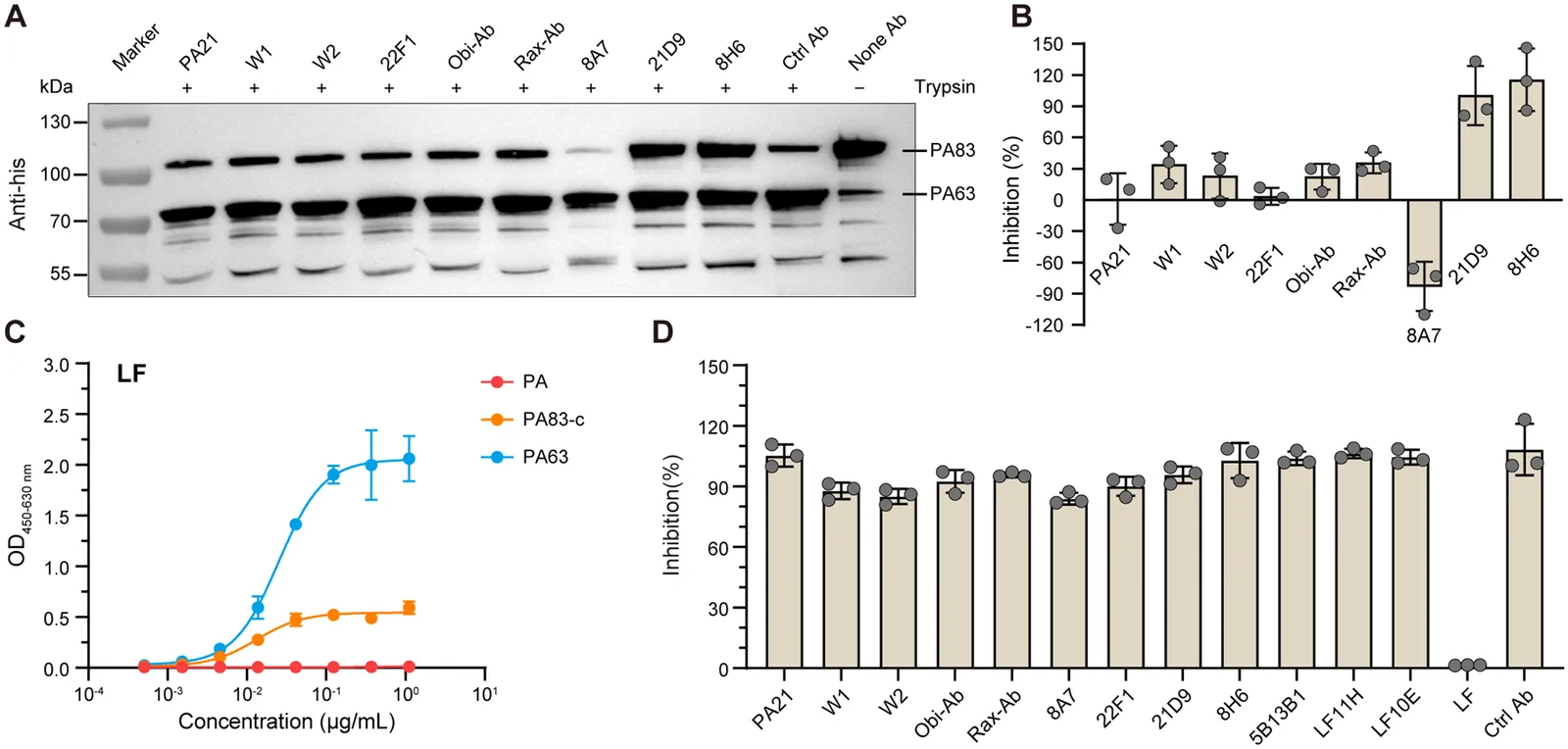
Antibody blockade of PA precursor cleavage and LF assembly. **(A, B)** Western blot analysis of the effect of antibodies on PA proteolytic processing. PA83 band intensities were quantified by ImageJ and normalized to the no‑antibody control. Data in (B) are presented as mean ± SD (*n* = 3). **(C)** Binding curves of biotinylated LF to different forms of PA determined by ELISA. Data are presented as mean ± SD (*n* = 3). **(D)** ELISA assessment of antibody blocking of biotinylated LF binding to PA63. Experimental OD values were normalized to the isotype control.

Next, the effect of antibodies on PA proteolytic processing was analyzed. Compared with the control antibody group, incubation with 21D9 or 8H6 led to a significant accumulation of uncleaved PA83 (Fig 5A and 5B). In contrast, consistent with previous observations [24], 8A7 promoted cleavage, resulting in almost complete digestion of the PA83 substrate, but the PA63 generated did not accumulate (likely due to rapid degradation or altered detection). Neither 22F1 nor the receptor-blocking antibodies (group A) exhibited any blocking or promoting effect on proteolytic processing, as the amounts of PA83 and PA63 did not differ significantly from those in the control group. In line with published data [33], LF efficiently bound only PA63 oligomers (Fig 5C). We therefore examined whether 22F1 blocks the binding of LF to PA63. The results showed that none of the PA or LF antibodies tested blocked this interaction (Fig 5D).

### 22F1 recognizes a previously unreported epitope on PA that is partially shielded by PA20

**Fig 6:**
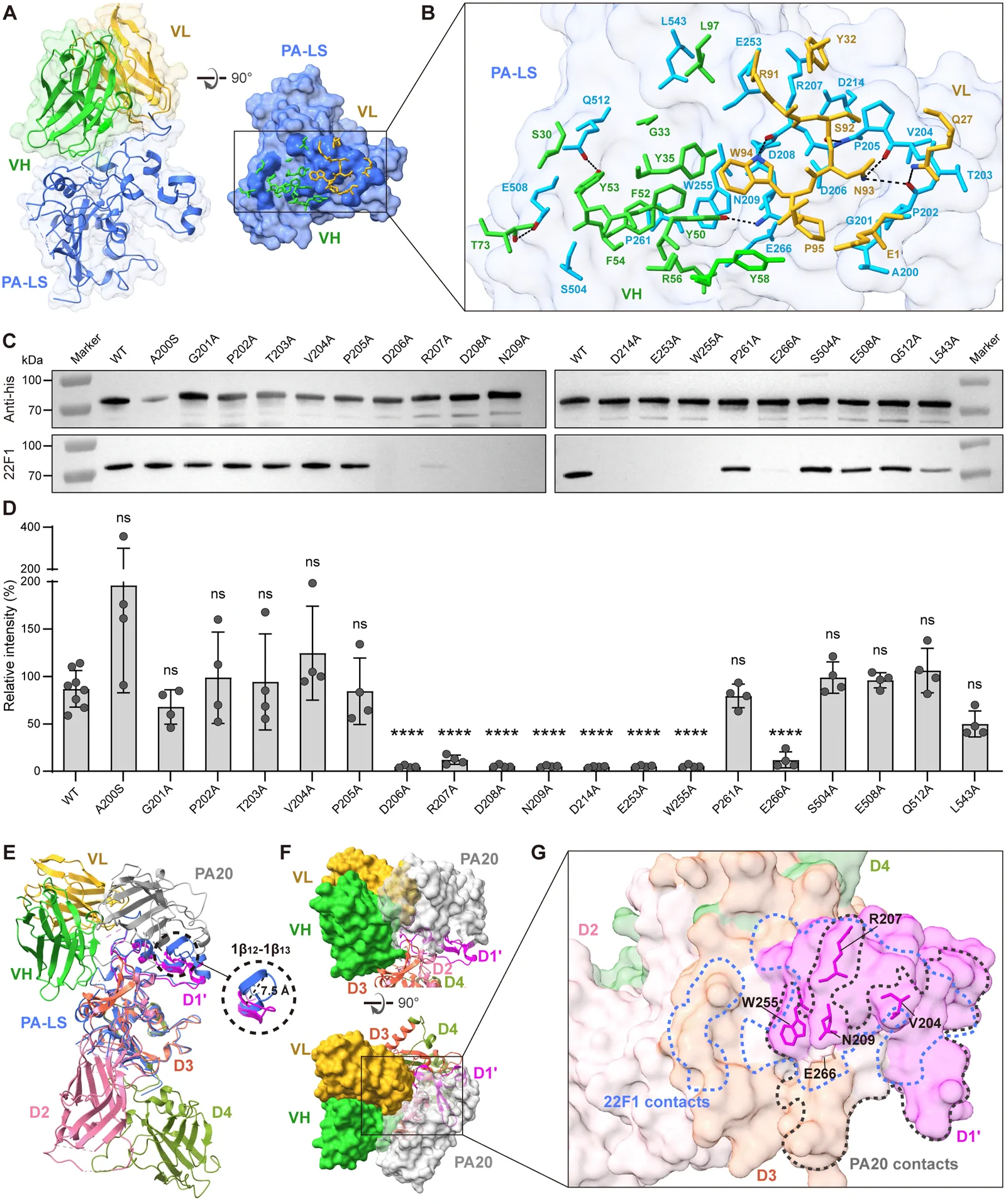
Cryo-EM structure of the 22F1 Fab in complex with PA83-c.

To elucidate the structural basis of 22F1 recognition, the three-dimensional structure of the 22F1 Fab in complex with PA83-c was determined by cryo-electron microscopy (S1 Fig and S1 Table). After multiple rounds of 2D and 3D classification, 121,316 particles from two independent datasets were selected for 3D reconstruction. Local refinement was performed at the antigen-antibody binding interface, yielding a 3D density map at a resolution of 3.06 Å (Fig 6A). The locally refined portion of PA was designated PA-LS. At the antigen-antibody interface, 19 amino acid residues of 22F1 (11 from the heavy chain and 8 from the light chain) interact with 19 residues of PA, covering an epitope area of approximately 930.8 Å² (Fig 6B). The heavy chain contributes predominantly through CDRH2 (6 of its 11 interface residues), whereas the light chain engages mainly via CDRL3 (5 of its 8 interface residues). The interaction network includes eight hydrogen bonds and two salt bridges (R56^CDRH2^ Nη2 – E266^PA^ Oε1; R91^CDRL3^ Nη2 – E253^PA^ Oε1). Although the light chain contributes fewer interface residues (8), it participates in interactions with 11 PA residues and provides most of the hydrogen bonds (5 out of 8), suggesting a dominant role in antigen recognition.

To identify critical epitope residues for 22F1, single-point mutants of PA63 were generated, and antibody binding was assessed by Western blotting (Fig 6C and 6D). Among the 19 interface amino acids, mutations of eight residues (D206, R207, D208, N209, D214, E253, W255, and E266) significantly reduced 22F1 binding. To understand the structural context of these critical residues, we superimposed PA-LS onto full-length PA83, which revealed that the 22F1 epitope is mainly located in domain D1’ (168–258 aa) of PA83 (13 out of 19 residues), followed by D3 (4 out of 19 residues), with the remaining two residues (P261, E266) situated in D2. Antibody binding induced a modest conformational change in PA-LS, with a root-mean-square deviation of 1.049 Å over 209 matched atom pairs (Fig 6E). The main conformational changes were concentrated in the 1β_12_–1β_13_ hairpin, which flipped upward as a whole, with an average displacement of 3.3 Å for 14 residues (T222–W235) and a maximum displacement of 7.5 Å. Notably, this 1β_12_–1β_13_ hairpin corresponds to the region with the largest movement of domain D1’ during PA pore formation [6]. Further analysis showed clear steric hindrance between the variable region of 22F1 and PA20, and the footprint of the antibody on PA63 largely overlapped with the region occupied by PA20 (Fig 6F and 6G). The overlapping region contains five shared amino acids (V204, R207, N209, W255, E266), four of which (except V204) were identified as critical residues for 22F1 binding. These findings explain both the inability of 22F1 to bind PA83 and its relatively lower binding signal to PA83-c (which contains PA63-PA20 complexes) (Fig 1D).

### 22F1 achieves ultrapotent neutralization via dual steric hindrance that blocks both additional antibody binding and PA63 oligomer assembly

**Fig 7:**
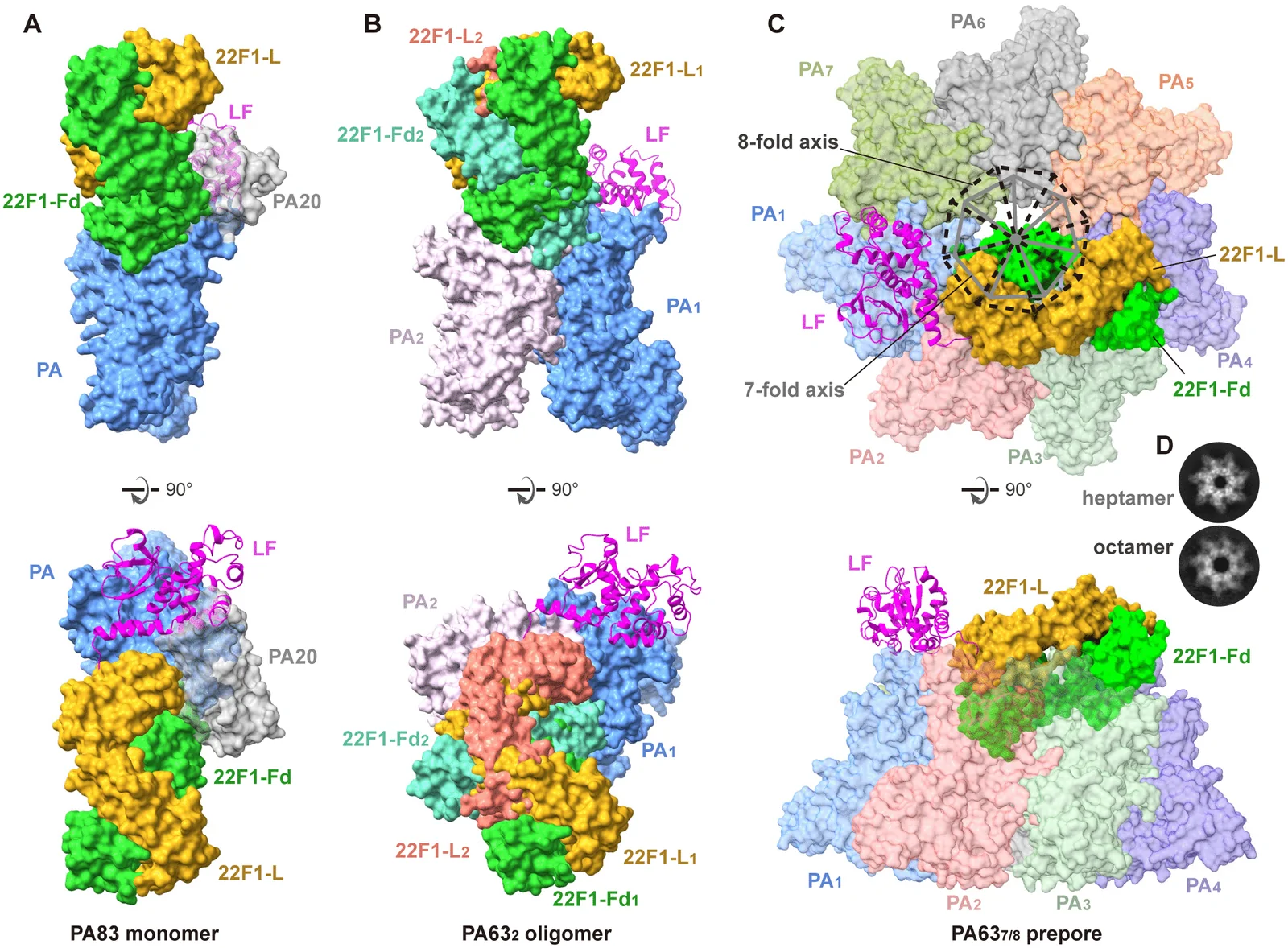
Structural superimposition of the 22F1 Fab‑PA‑LS complex and LF onto different PA structures. The following structures were used for superimposition: **(A)** the PA83 monomer (PDB ID: 1ACC), **(B)** a two‑protomer unit derived from the PA63 prepore (PDB ID: 3KWV), and **(C)** the PA63 prepore (PDB ID: 3J9C). Fd denotes the antibody VH and CH1 domains. In panel (C), the light grey solid line and dark grey dashed line represent seven‑fold and eight‑fold axes, corresponding to heptameric and octameric prepore symmetries, respectively. All molecules are rendered as molecular surfaces, with LF shown as a ribbon diagram. Colors are as indicated in the text labels. **(D)** Representative 2D class averages of heptameric and octameric PA prepores, as obtained during cryo‑EM structure determination. No density corresponding to 22F1 Fab was observed on the prepore oligomers.

To further elucidate the neutralization mechanism of 22F1, we performed a structural superposition of the 22F1 Fab and LF onto PA83 (PDB ID: 1ACC), a two-protomer structural unit (PA63_2_ oligomer) derived from the PA63 octamer (PDB ID: 3KWV), and the PA63 prepore (PDB ID: 3J9C) (Fig 7). At the PA monomer level, the epitopes of both 22F1 and LF are partially shielded by the PA20 domain (Fig 7A), consistent with the observation that neither binds efficiently to PA83 (Figs 1D and 5C). However, the epitopes of 22F1 and LF show almost no overlap, which aligns with the experimental finding that 22F1 does not block LF binding to PA63 (Fig 5D). In the dimeric interface, the 22F1 Fab approaches the interface boundary at the apex of the adjacent protomer from a nearly perpendicular orientation (Fig 7B). Due to severe steric hindrance between two 22F1 Fabs, binding of a single 22F1 Fab to one protomer prevents a second Fab from binding to the adjacent protomer. Further analysis revealed that the epitope recognized by the 22F1 Fab is located on the inner face of the PA prepore (Fig 7C). Binding of a single Fab occupies more than half of the space within the heptameric or octameric pore, thereby severely hindering the assembly of additional PA63 protomers. Moreover, once the prepore has assembled, the pore can no longer accommodate even a single 22F1 Fab. This conclusion is supported by cryo-EM structural evidence, as no Fab density was observed in the 2D class averages of the preformed prepore (S2 Fig). Thus, binding of the 22F1 Fab to any one of the PA63 protomers is sufficient to block prepore assembly. This mechanism provides a robust explanation for the exceptionally potent toxin-neutralizing activity of 22F1.

## Discussion

**Fig 8:**
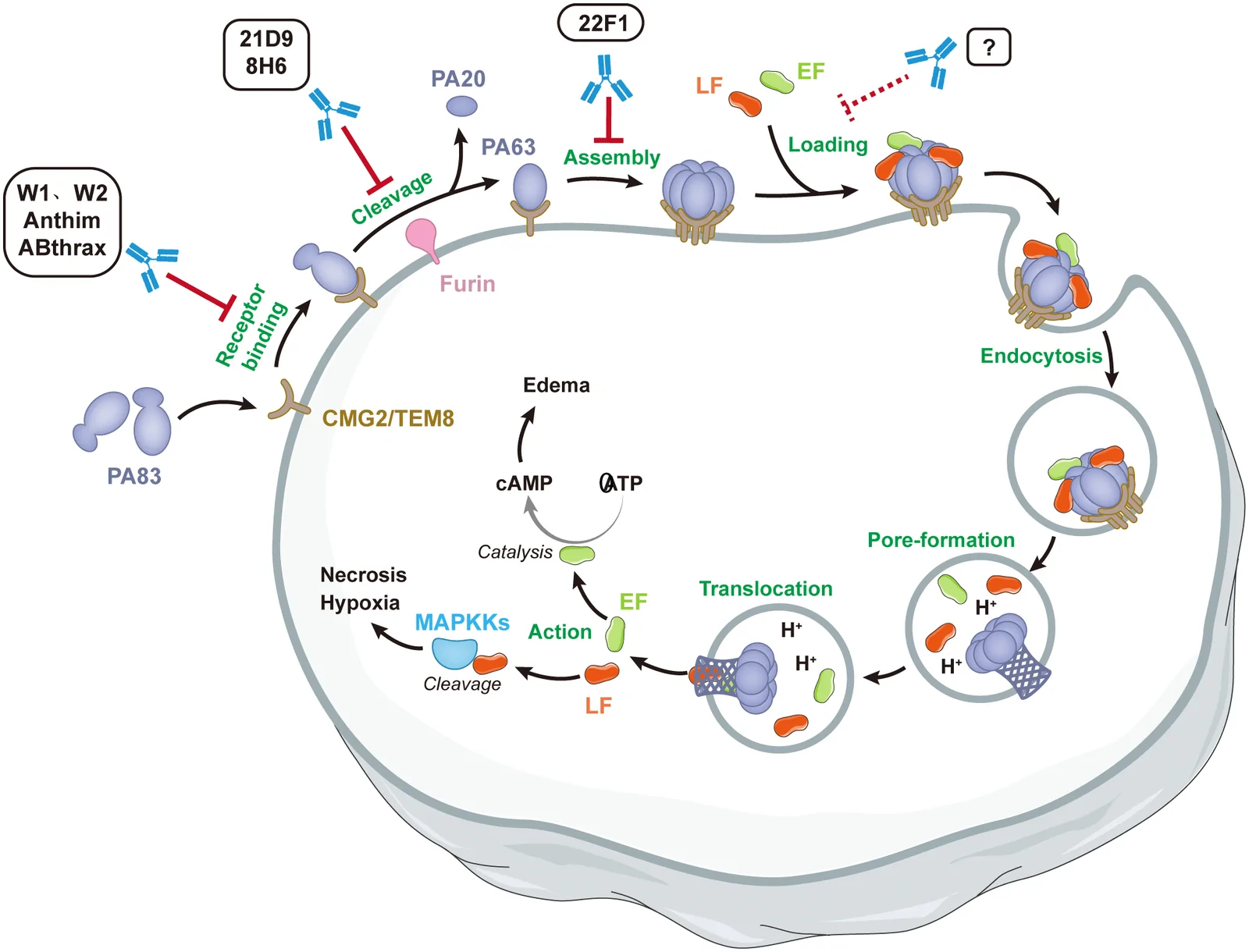
Schematic representation of anthrax toxin pathogenesis and antibody neutralization. The ultrapotent neutralization by 22F1 is mediated by steric hindrance that blocks PA63 oligomer assembly.

In this study, we characterized a human anti-PA antibody, 22F1, which exhibits a distinct binding pattern and exceptionally potent neutralization of anthrax lethal toxin. Unlike most neutralizing anti-PA antibodies that block receptor binding (e.g., raxibacumab, obiltoxaximab, W1, W2), interfere with PA proteolytic activation (e.g., 21D9), or inhibit LF/EF binding, 22F1 operates via a previously unrecognized mechanism: it binds to the D1’ domain of PA63 at an epitope that becomes accessible only after PA83 cleavage (Fig 8). Structural analysis revealed that the 22F1 epitope is partially shielded by PA20 in full-length PA83, explaining its negligible binding to the precursor. More importantly, in the context of PA63 homooligomers assembly, the epitope lies at the interface between adjacent protomers and is oriented such that binding of a single 22F1 Fab to one protomer creates severe steric hindrance, simultaneously preventing further 22F1 Fab binding to adjacent protomers and oligomerization of PA protomers, thereby blocking prepore formation. This “single-hit” blocking mechanism accounts for its sub-nanomolar IC₅₀ (0.027 nM) and superior in vivo protection with an antibody-to-toxin molar ratio as low as 1:7.5 to 1:15.

The potent activity of 22F1 raises an intriguing question: why have such high-efficacy antibodies not been more widely reported? One explanation is that traditional screening strategies often favor antibodies that bind strongly to full-length PA83, inadvertently excluding those that recognize cleavage-dependent epitopes. Notably, multiple studies have questioned the clinical benefits of the two approved monoclonal antibodies and AIG‑IV [34–37]. These controversies regarding efficacy are likely related to the relatively low toxin neutralization potency of these antibodies—in our observation, 22F1 exhibits up to several orders of magnitude higher neutralizing activity. This comparison underscores the substantial therapeutic advantage of discovering ultrapotent antibodies like 22F1, which can achieve efficacy at doses far below those of current standards. Our findings suggest that post-cleavage PA63 harbors unique neutralization-relevant sites that remain underexplored. More broadly, the concept of targeting an epitope at the inter-protomer interface to achieve substoichiometric neutralization—as exemplified by 22F1—may provide a new paradigm for the development of therapies against other pore-forming proteins that undergo an activation–oligomerization–pore formation cascade, such as *Clostridium perfringens* epsilon toxin [38], *Vibrio cholerae* cytolysin [39], perforin [40], pneumolysin [41], mixed lineage kinase domain-like pseudokinase [42], and gasdermins [43].

From a translational perspective, 22F1 offers several clear advantages. Its thermal stability is favorable for manufacturing and storage. The extremely low effective dose (2.5–5 μg per rat) suggests that clinical doses could be significantly lower than those of approved products, potentially reducing costs and adverse reactions. Moreover, 22F1 exclusively recognizes cleaved PA, avoiding consumption by free, non-toxic PA83 precursor—a pharmacokinetic advantage over antibodies that also bind the inactive form. However, several limitations must be acknowledged. First, our in vivo protection model employed a toxin challenge instead of a live *B. anthracis* spore challenge. Although the FDA Animal Rule permits efficacy studies based on toxin challenge, the presence of bacterial replication and capsule-mediated immune evasion may alter the therapeutic requirements. Second, we did not evaluate 22F1 in combination with antibiotics or other immune modulators, which would be essential for clinical translation. Third, although certain anti‑PA antibodies have been shown to delay spore germination and enhance macrophage phagocytosis and sporicidal activity in vitro [44], 22F1—given its inability to bind PA83—may lack the capacity to mediate such anti‑spore effects during the early stage of anthrax infection. Fourth, although 22F1 is of human origin, its immunogenicity risk has not been directly assessed.

In this study, several previously reported PA‑binding antibodies (e.g., PA21 [17], LF11H [31]) exhibited good antigen‑binding ability but failed to neutralize LT, whereas the antibody LF10E, previously described as strongly neutralizing [31], showed almost no binding to LF in our hands—possibly due to differences in experimental models and issues with its preparation. Another notable finding is that some non‑neutralizing antibodies (e.g., 8A7) conferred partial protection in vivo despite lacking in vitro activity, although for other antibodies tested the survival benefit did not reach statistical significance. This phenomenon, also observed in other viral and bacterial systems, suggests that Fc‑mediated effector functions contribute to protection [45]. Whether 22F1 might also benefit from such mechanisms warrants further investigation. Finally, while antibody cocktails and bispecific antibodies have shown promise, our data demonstrate that a single well-designed monoclonal antibody can achieve superior neutralization potency, highlighting the value of discovering antibodies with atypical epitopes and unique mechanisms. Future work should include spore challenge studies and explore the potential of 22F1-derived bispecific formats targeting both PA and the capsule.

## Materials and methods

### Ethics statement

All animal experiments were conducted at Sino Animal Science and Technology Development Co., Ltd., and were approved by the Institutional Animal Care and Use Committee (Approval No. 20250213YZA-3R).

### Plasmid construction

The genes encoding the variable regions of PA21, W1, W2, 21D9, LF11H, LF10E, 5B13B1, 8A7, and 8H6 (each fused to human IgG1 constant region), the 22F1 VH-CH1 domain for Fab fragment, and PA63 (197–764aa) with a C‑terminal 6 × His tag were codon-optimized, synthesized (Sangon Biotech), and cloned into pcDNA3.1 (for antibodies and Fab) or pET21a (for PA63), respectively.

### Generation of different PA forms

PA83 was digested with trypsin (1 mg/mL, Sigma, T1426) at 37°C for 30 min, and the reaction was terminated with trypsin inhibitor (2 mg/mL, Sigma, T9777) at a PA:trypsin:inhibitor mass ratio of 1000:1:10. The digest was concentrated to ~0.6 mL using a 10 kDa ultrafiltration unit (Merck Millipore, UFC5010BK) and separated on a Superdex 200 10/300 GL column (GE Healthcare). UV peaks were collected, and purity was assessed by SDS-PAGE under reducing conditions (5 μg/lane) on 4–12% Bis-Tris gels (GenScript, M41210C) run in Tris-MOPS-SDS buffer (GenScript, M00138) at 80 V for 10 min followed by 150 V for 40 min. Gels were stained with eStain L1 (GenScript) and imaged with an iBright FL1500 (Invitrogen). Purified PA proteins were concentrated, quantified, aliquoted, and stored at -80°C.

### Antibody production

For in-house antibody production, Expi293F cells were transiently transfected with antibody heavy and light chain genes using the ExpiFectamine 293 Transfection Kit (Thermo Fisher Scientific, A14526) according to the manufacturer’s protocol. The transfected cells were cultured at 37°C with 8% CO_2_ and agitated at 125 rpm for 4–5 days. The culture supernatant was clarified by sequential centrifugation (800 × *g*, 15 min, 4°C; then 3500 × *g*, 15 min, 4°C) and filtered through a 0.22 μm filter (PALL, 4612). Purification was performed on an AKTA Pure 150 system (GE Healthcare) using a 5 mL rProtein A affinity column (Cytiva). Binding buffer was PBS (pH 7.4), elution buffer was 0.1 M glycine (pH 3.0), and neutralization buffer was 1 M Tris‑HCl (pH 9.0). Purified antibodies were concentrated and buffer‑exchanged into PBS using a 30 kDa ultrafiltration unit (Merck Millipore, UFC903096), then quantified, aliquoted, and stored at -80°C. The following antibodies were purchased directly from commercial sources: obiltoxaximab (MedChemExpress, HY‑P9932) and raxibacumab (MedChemExpress, HY‑P9957).

### Binding activity assay

96‑well plates (Corning, 9018) were coated with PA proteins (1 μg/mL in 50 mM carbonate buffer, pH 9.6) at 4°C overnight. After washing with PBST (PBS containing 0.05% Tween 20), wells were blocked with 100 μL of 2% BSA in PBS at 37°C for 1 h. Serially diluted antibodies (starting at 5 μg/mL, 3‑fold dilution) were added and incubated at 37°C for 1 h. After washing, HRP‑conjugated goat anti‑human IgG Fc (Abcam, ab97225, 1:10000) was added and incubated at 37°C for 1 h. TMB substrate (Solarbio, PR1200, 100 μL) was added for 5–6 min at 25°C, and the reaction was stopped with 50 μL stop solution (Solarbio, C1058). Absorbance was read at 450 nm and 630 nm using a SpectraMax ABS plate reader (Molecular Devices), and the difference (OD_450_–OD_630_) was calculated. EC₅₀ values were obtained by fitting a four‑parameter dose‑response curve in GraphPad Prism.

### Competition and LF blocking assays

Plates were coated with PA63 (1 μg/mL). Unlabeled antibodies or LF protein (20 μg/mL) were added together with an equal volume of biotinylated antibody or biotinylated LF (0.2 μg/mL) and incubated at 37°C for 1 h. After washing, streptavidin‑HRP (Abcam, ab7403, 1:10000) was added and incubated at 37°C for 1 h. The remaining steps were the same as described above.

### In vitro toxin neutralization assay

J774A.1 cells were seeded in 96‑well plates (4 × 10⁴ cells/well) and cultured overnight at 37°C, 5% CO_2_. In separate plates, serially diluted antibodies (starting at 2 μg/mL, 3‑fold dilution) were mixed with PA and LF (each at a final concentration of 150 ng/mL). Toxin‑only and medium‑only controls were included. After incubation at 37°C for 1 h, the cell supernatant was removed, and 100 μL of the toxin‑antibody mixture was transferred to each well. Cells were incubated for 4 h, then the supernatant was replaced with 100 μL of MTT solution (1 mg/mL, Sigma, M2128) and incubated for another 4 h. The supernatant was discarded, and formazan was dissolved in 100 μL DMSO (Solarbio, D8371). Absorbance was measured at 570 nm with a reference wavelength of 630 nm. Cell viability (%) was calculated as (OD_test_ – OD_toxin_) / (OD_medium_ – OD_toxin_) × 100%. IC_50_ values were calculated by fitting a four‑parameter dose‑response curve in GraphPad Prism. For synergistic neutralization assay, antibody pairs (each at 10 ng/mL) were mixed with toxins (each at 150 ng/mL), the remaining steps were the same as described above.

### Bio‑layer interferometry

Measurements were performed on a Gator Bioanalysis System (Gator Bio). Anti-Human Fc probes (Gator, 20–5036) were equilibrated in PBS for 180 s, loaded with 20 nM 22F1 for 120 s, and baseline was established for 60 s. Association with serially diluted PA83‑c (200–6.25 nM, 2‑fold dilution) was monitored for 240 s, followed by dissociation in buffer for 480 s. A reference sensor loaded with antibody only was used for background subtraction. Data were fitted globally to a 1:1 Langmuir binding model using Gator Part 11 Software (Gator Bio) to calculate the kinetic parameters.

### Proteolytic processing blocking assay

PA83 was pre‑incubated with antibodies at a 1:1.5 molar ratio in PBS at 37°C for 1 h, followed by trypsin digestion as described above. The 15 μL samples (containing ~200 ng of the initial substrate PA83) were then separated by reducing SDS‑PAGE and transferred to nitrocellulose membranes. Membranes were blocked with 5% skim milk at 25°C for 1 h, washed with PBST, and incubated overnight at 4°C with 5 μg/mL of anti‑PA83/PA63 antibody (Alpha Diagnostic International, PA12‑M) as the primary antibody. After washing, HRP‑conjugated anti‑mouse IgG (Abcam, ab131368, 1:5000) was added for 1 h at 25°C. Signals were developed using chemiluminescent substrate (Merck Millipore, WBKLS0100) and imaged with an iBright FL1500 (Thermo Fisher Scientific). Relative blocking percentage was calculated as [1 – (gray value with antibody / gray value without antibody)] × 100%.

### Differential scanning calorimetry

Thermal stability was measured using a MicroCal PEAQ-DSC (Malvern). After a 40 min instrument warm-up, the cells were washed with cleaning solution and water. PBS and the 22F1 antibody were degassed under vacuum (635 mmHg, 15 min). Following removal of residual liquid, 800 μL of degassed PBS was added to the cells, and any air bubbles were removed. A baseline scan was then performed from 25°C to 100°C at 3 atm. The system was cooled to 30°C, the pressure was released, and the cells were cleaned again. Subsequently, 800 μL of degassed PBS containing 22F1 antibody (1 mg/mL) was loaded, and the scan was repeated. Data were fitted to a two-state scaled model using MicroCal PEAQ-DSC software v1.64 to obtain the Tm values.

### Receptor blocking assay

One day before transfection, 6 × 10^6^ HEK293T cells were seeded in T75 flasks and cultured in DMEM at 37°C with 5% CO_2_. When cells reached approximately 80% confluence, transfection was performed using Lipofectamine 3000 Transfection Reagent (Thermo Fisher Scientific, L3000015) according to the manufacturer’s protocol. Briefly, 15 μg of CMG‑2 or TEM‑8 receptor plasmid was mixed with Opti-MEM I Reduced Serum Medium (Thermo Fisher Scientific, 31985070) and combined with Lipofectamine 3000. The mixture was incubated at 25°C for 15 min and then added to the cells. After 6 h, the medium was replaced with complete DMEM. After 36 h, cells were harvested, washed, and pelleted (600 × *g*, 5 min). Approximately 1 × 10^5^ cells per tube were washed with 3 mL PBS (600 × *g*, 5 min). PA83 was labeled with Alexa Fluor 647 (Invitrogen, A20173). A 100 μL aliquot of the labeled PA (1 μg/mL) was pre‑incubated with antibodies (4 μg/mL) at 37°C in the dark for 1 h. Then, 100 μL of the mixture was added to each tube and incubated at 25°C for 1 h. After two washes with PBS, cells were resuspended in 200 μL PBS and analyzed on a FACSCanto II flow cytometer (BD Biosciences) (20,000 events per tube). Relative binding (%) was calculated as (number of positive cells with antibody / number of positive cells with irrelevant antibody) × 100%.

### Anthrax toxin challenge in Fischer 344 rats

Fischer 344 rats (56–62 days old, male/female 1:1) were purchased from Charles River Co., Ltd. (Beijing). Animals were acclimated for one week prior to use.

Rats were randomly assigned to groups (*n* = 6 per group). Each injection mixture contained PA (20 μg), LF (10 μg), and either 20 μg of antibodies or graded doses (20, 10, 5, 2.5, 1.25, or 0 μg) of the 22F1 antibody, diluted to a final volume of 200 μL with PBS. The mixtures were administered intravenously via the tail vein. Survival and body weight were monitored for 7 days. Moribund rats were humanely euthanized during the observation period, and all surviving rats were euthanized at the end of the experiment (day 7) in accordance with institutional ethical guidelines.

### Electron microscopy, image processing, and 3D reconstruction

*CryoEM grid preparation and data acquisition.* A total of 70.0 μL purified PA protein at the concentration of 1.76 mg/mL was incubated with 50.0 μL 22F1 Fab at the concentration of 1.87 mg/mL at a 1:1.3 molar ratio on ice for 40 min for the next step of size exclusion chromatography. After centrifugation (13,500 × *g*, 4 °C for 5 min), 100 μL supernatant was injected to GE micro Akta using Superdex 200 column. The peak fraction was applied for cryo-EM grid preparation.

An aliquot of 4 μL protein sample of PA_22F1 Fab complex was applied onto a glow-discharged 300 mesh grid (Quantifoil Au R1.2/1.3), some grids were supported with a thin layer of GO (Graphene Oxide) or RGO (Reduced Graphene Oxide), blotted with filter paper for 4.5 s and plunge-frozen in liquid ethane using a Thermo Fisher Vitrobot Mark IV. Cryo-EM micrographs were collected on a 300kV Thermo Fisher Krios G4 electron microscope equipped with a Falcon 4 direct detection camera and a Selectris image filter (GIF: a slit width of 10 eV). The micrographs were collected at a calibrated magnification of ×165,000, yielding a pixel size of 0.75Å at a super resolution counting mode. In total, 12,751 micrographs were collected at an accumulated electron dose of 50e^-^Å^-2^s^-1^ on each micrograph that was fractionated into a stack of 32 frames with a defocus range of −1.0 μm to −2.0 μm.

*EM data processing.* Beam-induced motion correction was performed on the stack of frames using MotionCorr2 [46]. The contrast transfer function parameters were determined by CTFFIND4 [47]. A total 12,751 good micrographs were selected for further data processing using cryoSMART. Particles were auto-picked by the Auto-picking program in cryoSMART, followed by 4 rounds of reference-free 2D classifications. Next, 921,739 particles were selected from good 2D classes and were subjected to two rounds of 3D classification using a reconstruction of the PA-22F1 Fab complex as a starting model. Three converged 3D classes with a feature containing PA and 22F1 Fab were selected for a final round of 3D refinement. After a round of final 2D classification, 121,316 particles from those three 3D classes shows the highest resolution feature of the epitope of PA and 22F1 Fab were selected for 3D refinement, yielding a final reconstruction at a global resolution of 3.06 Å based on the gold-standard Fourier shell correlation criterion at FSC = 0.143. The local resolution was then calculated on the final density map.

*Model building and refinement*. The model of PA-22F1 Fab complex was built by fitting the model of structure of PA and 22F1 Fab (predicted by AlphaFold2) into the density map using UCSF Chimera [48,49], followed by a manual model building in COOT [50].

### Identification of critical epitope residues

Single‑point mutations were introduced into wild‑type PA63 plasmids using the Q5 Site‑Directed Mutagenesis Kit (New England Biolabs, E0552S). Plasmids were extracted using the TIANprep Plasmid Midi Kit (TIANGEN, DP106‑02) and verified by sequencing. Mutant plasmids were transformed into competent *E. coli* BL21 cells (TIANGEN, CB105‑02). Single colonies were grown in LB medium containing kanamycin (50 μg/mL) at 37°C to an OD_600_ of approximately 0.6, and protein expression was induced with 0.5 mM IPTG (Amresco, 0487) at 15°C for 24 h. Cells were harvested by centrifugation, lysed by sonication, and the soluble fraction was purified by nickel affinity chromatography, followed by ultrafiltration concentration and quantification.

Equal amounts of PA63 mutants (~200 ng) were separated by reducing SDS‑PAGE, transferred to membranes, and probed with either HRP‑conjugated anti‑His antibody (Abcam, ab1187, 1:2500) or 22F1 antibody (0.5 μg/mL) followed by HRP‑conjugated secondary antibody (Abcam, ab97225, 1:10000). Signals were detected as described above. Relative binding (%) was calculated as (22F1 signal gray value / anti‑His signal gray value) × 100%.

### Sequence and structural analysis

The Kabat numbering of the 22F1 VH and VL amino acid residues displayed in the cryo-EM structural panels was annotated using abYsis v3.4.1 (http://www.abysis.org/abysis/) [51]. Germline gene assignment of the antibody was performed using the IMGT/V-QUEST program v3.6.3 (https://www.imgt.org/IMGT_vquest) [52]. Structural superpositions, surface burial measurements, and analyses of antibody–antigen interacting residues and hydrogen bonds were carried out using ChimeraX v1.10.1 [53].

### Statistical analysis

All quantitative experiments were independently repeated at least three times, and data are presented as mean ± standard deviation (SD). EC₅₀ values from binding assays and IC₅₀ values from neutralization assays were determined by fitting the data to a four‑parameter curve. The log‑rank (Mantel‑Cox) test was used for animal challenge studies. All statistical analyses were performed using GraphPad Prism v 10.1.2. Significance levels were defined as *P < 0.05, **P < 0.01, ***P < 0.001, and ****P < 0.0001. Further details are provided in the corresponding figure legends.

## Supporting information

S1 Table Cryo-EM data collection, refinement and validation statistics. (DOCX)

S1 Fig Cryo electron microscopy data collection and processing of the 22F1 Fab in complex with PA-LS. (A) Workflow of the data processing procedure. (B) Gold standard Fourier shell correlation (FSC) curves of the Ab-Ag complex density map. (C) Local resolution of the Ab Ag complex model.
(TIF)

S2 Fig Representative 2D class averages of the PA63 pre-pore structure. (TIF)

S1 Raw Gel (A) Uncropped reducing SDS-PAGE gel corresponding to Fig 1B, showing chromatographic peak fractions. (B) Uncropped western blot membrane corresponding to Fig 5A, showing the effect of antibodies on PA proteolytic processing. (C) Uncropped western blot membrane corresponding to Fig 6C, used for identification of key epitope residues. (DOCX)

S1 Data Source Data for Figures. (XLSX)
