# Correction: Ultrapotent neutralization of *Bacillus anthracis* toxin by a human antibody via blockade of PA oligomerization

**DOI:** 10.1371/journal.ppat.1014594

**Published:** 2026-09-15

**Authors:** Qi Wang, Ting Fang, Zhenwei Song, Xiaoyan Huang, Sijun He, Tianfu Li, Jianmin Li, Xiangyang Chi, Pengfei Fan, Changming Yu

There are errors in the legend of Fig 3. Please see the correct Fig 3 legend here.

The legend of Fig 6 was omitted from the article. The legend have been provided here:

**Fig 3:**
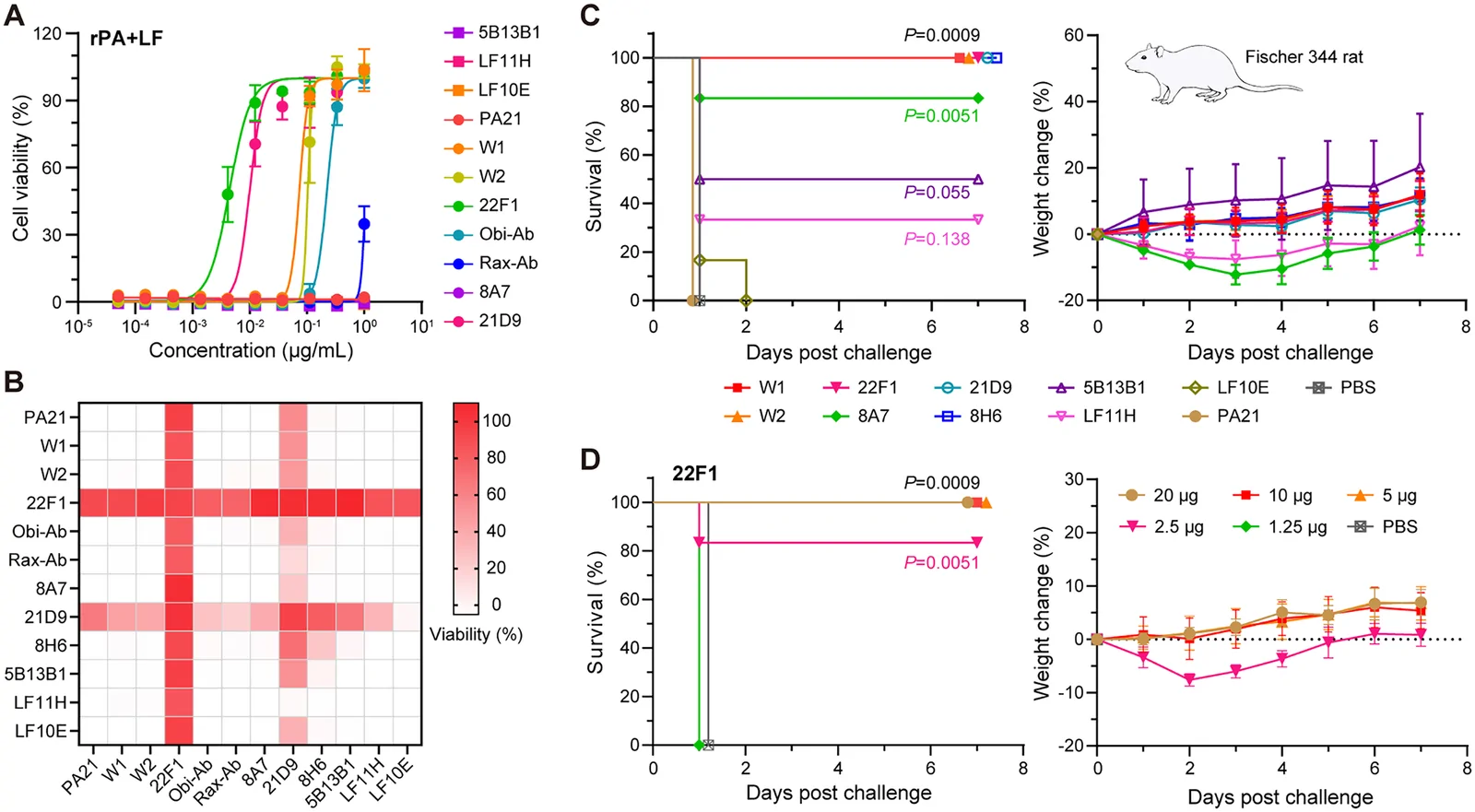
In vitro and in vivo neutralization of anthrax lethal toxin by antibody 22F1. **(A)** Neutralization activity of the indicated antibodies against lethal toxin (LT) in J774A.1 cells. Data are presented as mean ± SD. **(B)** Synergistic neutralization assay. Antibody pairs were each used at a final concentration of 10 ng/mL. **(C)** Survival and body weight changes of Fischer 344 rats (n = 6) after intravenous (tail vein) injection of antibody (20 μg) mixed with toxins (PA 20 μg + LF 10 μg). Survival was monitored for 7 days. P values were determined by the Mantel–Cox log-rank test. *P < 0.05, **P < 0.01, ***P < 0.001. **(D)** Dose-dependent protection of 22F1. Rats received fixed toxin doses (PA 20 μg + LF 10 μg per rat) combined with 22F1 at 20, 10, 5, 2.5, 1.25, or 0 μg per rat (n = 6 per group). P values are the same as in (C).

**Fig 6:**
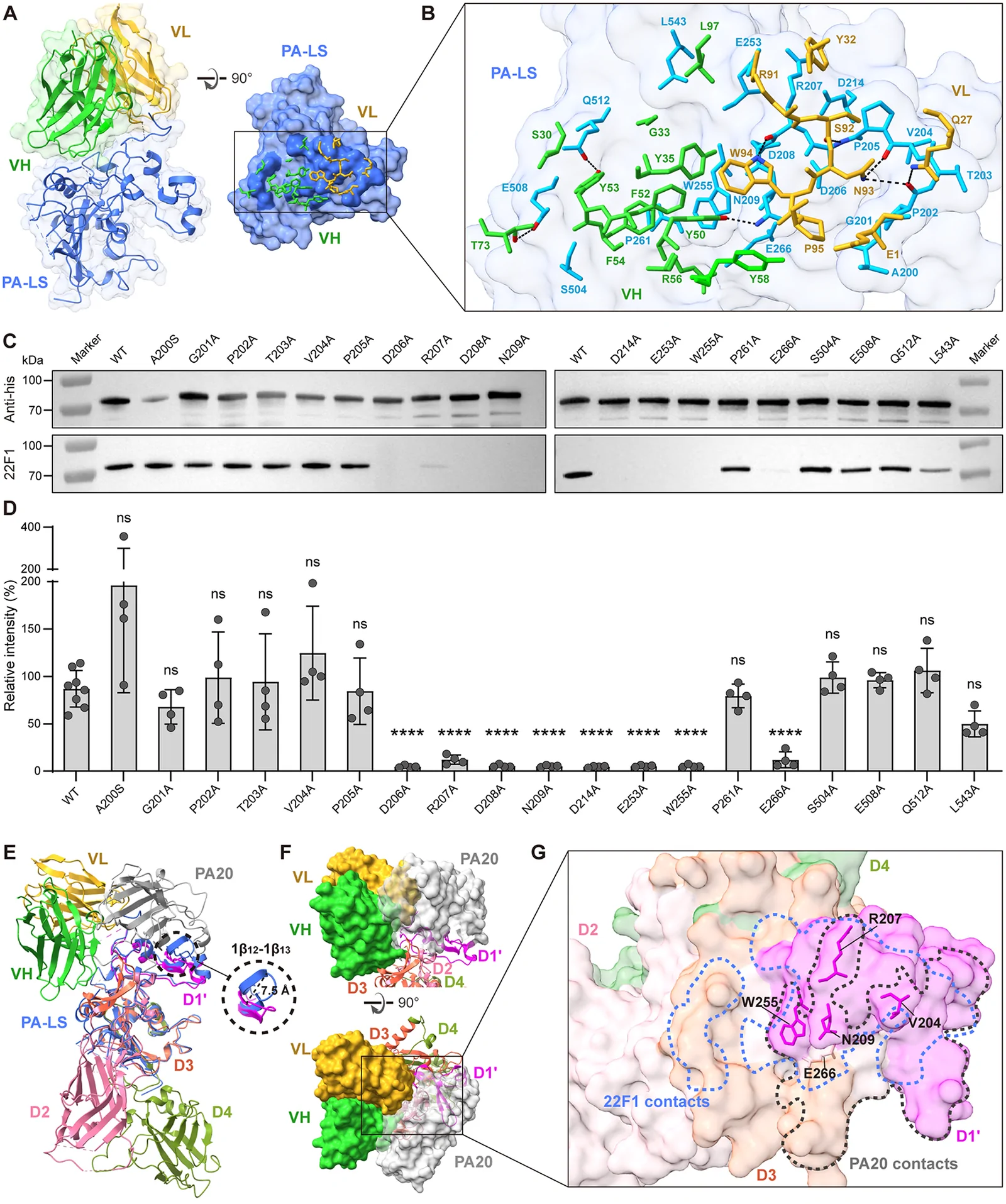
Cryo-EM structure of the 22F1 Fab in complex with PA83-c. **(A)** Front and top views of the cryo-EM structure of the 22F1 Fab in complex with PA83-c. The resolved portion of PA is designated PA-LS. PA-LS is colored in royal blue, and the VH and VL chains are colored in lime green and goldenrod, respectively. In the front view, 22F1 Fab and PA-LS are shown as ribbon diagrams beneath a transparent surface. In the top view, PA-LS is represented as a molecular surface, and the epitopes of 22F1 are highlighted. The interface residues of 22F1 are shown as stick representations. **(B)** Magnified view of the interface between 22F1 and PA-LS. PA-LS is shown as a transparent surface. Antigen and antibody residues within 4Å of the interface are depicted as stick representations, and hydrogen bonds are shown as broken black lines. The oxygen and nitrogen atoms involved in hydrogen bonds are colored in red and blue, respectively. **(C)** Identification of key epitope residues by western blotting. Equal amounts of wild-type or mutant PA63 were detected with anti-His antibody and 22F1 antibody, respectively. **(D)** Quantitative analysis of relative binding. Relative binding (%) was calculated as (22F1 signal gray value/ anti-His signal gray value) × 100%. Data are presented as mean ± SD (n = 4). A one-way ANOVA was used to compare the binding capacity of 22F1 to different PA63 mutants versus wild-type PA63; ns, P > 0.05; ****P < 0.0001. **(E)** Structural superposition of PA-LS and full-length PA83 (PDB ID: 1ACC). Molecules are shown as ribbon diagrams. 22F1-VH, 22F1-VL, PA-LS, and the PA20, D1′, D2, D3, and D4 domains of PA are colored in lime green, goldenrod, royal blue, gray, magenta, pale violet red, tomato, and olive drab, respectively. The structure highlighted by the black dashed circle is the 1β12-1β13 hairpin loop (T222–W235). **(F)** Steric hindrance analysis of the 22F1 variable region and PA20. 22F1-VH and 22F1-VL are shown as molecular surfaces, and PA-LS is shown as a transparent surface. **(G)** Footprint analysis of 22F1 (blue dashed line) and PA20 (black dashed line) on PA63. The shared PA63 contact residues are shown as stick representations.
