# Supplementary material for: Ultrapotent neutralization of Bacillus anthracis toxin by a human antibody via blockade of PA oligomerization
Source: PLoS Pathog. 2026 Aug 14;22(8):e1014523. doi: 10.1371/journal.ppat.1014523 (PMC13497254; doi:10.1371/journal.ppat.1014523)
Supplement: Supplementary file 1 [file ppat.1014523.s001.docx]

S1 Table. Cryo-EM data collection, refinement and validation statistics

|  | **PA-LS_22F1-Fab complex** |
| --- | --- |
| **Data collection and processing** |  |
| Magnification | 165k |
| Voltage (kV) | 300 |
| Electron exposure (e^–^/Å^2^) | 47.04 |
| Defocus range (μm) | -1.0~-2.0 |
| Pixel size (Å) | 0.75 |
| Symmetry imposed | C1 |
| Initial particle images (no.) | 6,113,368 |
| Final particle images (no.) | 121,316 |
| Map1 resolution (Å)  FSC threshold | 3.06  0.143 |
| Map resolution range (Å) | 2.6-6 |
|  |  |
| **Refinement** |  |
| Initial model used (PDB code) | predicted by ModelSmart |
| Model resolution (Å)  FSC threshold | 3.06  0.143 |
| Model resolution range (Å) | 2.6-6 |
| Map sharpening *B* factor (Å^2^) | 126.4 |
| Model composition  Protein residues | 685 |
| R.m.s. deviations  Bond lengths (Å)  Bond angles (°) | 0.003  0.609 |
| Validation  Clashscore  Poor rotamers (%) | 7.61  0.70 |
| Ramachandran plot  Favored (%)  Allowed (%)  Disallowed (%) | 95.08  4.92  0.00 |
