# Supplementary material for: Ultrapotent neutralization of Bacillus anthracis toxin by a human antibody via blockade of PA oligomerization
Source: PLoS Pathog. 2026 Aug 14;22(8):e1014523. doi: 10.1371/journal.ppat.1014523 (PMC13497254; doi:10.1371/journal.ppat.1014523)
Supplement: Supplementary file 2 [file ppat.1014523.s004.docx]

**A.**

B.

C.

**S1 Raw gel and blot images.**

(A) Uncropped reducing SDS-PAGE gel corresponding to Fig 1B, showing chromatographic peak fractions. (B) Uncropped western blot membrane corresponding to Fig 5A, showing the effect of antibodies on PA proteolytic processing. (C) Uncropped western blot membrane corresponding to Fig 6C, used for identification of key epitope residues.
